# Effect of population aging on pulmonary tuberculosis burden in Zhejiang Province, China: a population-based study

**DOI:** 10.3389/fpubh.2025.1647139

**Published:** 2025-10-29

**Authors:** Yuxiao Ling, Wei Wang, Songhua Chen, Yu Zhang, Qian Wu, Xinyi Chen, Kui Liu, Ke Yang, Dan Luo, Yang Li, Yiqing Zhou, Bin Chen, Jianmin Jiang

**Affiliations:** ^1^School of Public Health, Health Science Center, Ningbo University, Ningbo, Zhejiang, China; ^2^Department of Tuberculosis Control and Prevention, Zhejiang Provincial Center for Disease Control and Prevention, Hangzhou, China; ^3^Key Laboratory of Vaccine, Prevention and Control of Infectious Disease of Zhejiang Province, Hangzhou, China; ^4^School of Public Health, Hangzhou Medical College, Hangzhou, Zhejiang, China; ^5^School of Public Health, Hangzhou Normal University, Hangzhou, Zhejiang, China

**Keywords:** aging, demographic factors, incidence, prediction, pulmonary tuberculosis

## Abstract

**Background:**

China’s aging population poses a growing challenge to disease burden. This study aims to evaluate the impact of population aging on the incidence of pulmonary tuberculosis (PTB) in Zhejiang Province, China.

**Methods:**

PTB notified incidence data reported to the National Notifiable Diseases Reporting System from 2005 to 2022 were analyzed. The notified incidence from 2023 to 2035 was estimated using exponential regression models. The decomposition algorithm was applied to decompose and quantify the effects of population aging, population growth, and age-specific incidence on the PTB incidence.

**Results:**

Between 2005 and 2022, the notified incidence of PTB in Zhejiang Province declined from 93.5 per 100,000 to 33.99 per 100,000. The highest notified incidence was observed in those aged 60 years and older, with the population attributable fraction increasing from 18.76 to 27.92%. During this period, PTB cases decreased by 51.22% compared to 2005, driven by a 23.83% increase due to population growth, a 10.26% increase due to age structure, and an 85.31% decrease due to age-specific incidence. The contribution of age structure to the increase in the number of cases was −1.92, −1.69%, 3.28, and 10.60% in the age groups 0–19, 20–39, 40–59, and ≥60 years, respectively.

**Conclusion:**

Notified incidence of PTB in Zhejiang Province is expected to decrease. However, this decrease may be affected by demographic changes, particularly in older adults. Thus, strengthening public health measures and policies is crucial to achieving the End TB Strategy goal by 2035, emphasizing the need to enhance PTB screening in older adults.

## Introduction

Tuberculosis (TB) is a major global public health threat, with an estimated 10.8 million new cases and 1.25 million deaths worldwide in 2023. In China, there were approximately 741,000 new cases and 27,000 TB-related deaths in 2023, with an incidence of 52 per 100,000 (1).

Several factors increase the risk of TB reactivation and reinfection in older adults, including aging, comorbidities, and compromised immunity (2–5). Although cough, sputum production, weight loss, and fatigue/malaise are common in older adults (6), other symptoms are less common, such as fever, sweating, and hemoptysis (7). These atypical clinical features may complicate TB diagnosis, leading to treatment delays and diminished treatment effectiveness (7).

The risk of developing TB increases with age (8). More than 25% of all TB cases reported in the United States since 2017 occurred in older adults, who represent approximately 16% of the total population (4). In the Lao People’s Democratic Republic, a national survey revealed that TB prevalence was 15-fold higher among individuals aged 65 years and older compared to those under 25 (9). Moreover, the age-period-cohort analysis showed that TB prevalence increased in older age groups in China and India (10), indicating a shift in TB burden toward the older adults.

China transitioned into an aging society in 1999 and currently has the world’s largest older population (11). Projections estimate a further increase, with an anticipated 395 million people aged 65 years and older by 2050 (12). This demographic shift, coupled with longer life expectancy and TB reactivation, poses new challenges to TB prevention and control in China (13).

WHO’s End TB Strategy aims to reduce global TB incidence to 90% of 2015 levels by 2035 and eliminate TB by 2050. While China has made progress in reducing TB (14), the 2025 interim target remains distant. Notably, studies indicated that future declines in TB incidence and mortality in China were primarily influenced by disease reactivation and population aging rather than new transmissions (15).

Addressing the challenges posed by population aging is crucial for TB elimination. While previous studies have evaluated age-related changes in TB prevalence using incidence data, there is little knowledge about the effect of demographic shifts on disease burden. This study focused on Zhejiang Province, which has a well-developed economy and a relatively low prevalence of TB, making it a favorable setting to achieve the End TB Strategy goal. To gain a realistic understanding of trends and potential causes of pulmonary tuberculosis (PTB) epidemics, we evaluated the effect of demographic and epidemiological changes on PTB incidence. These data are essential to formulate targeted prevention and control measures.

## Materials and methods

### Study setting

This study was conducted in Zhejiang Province, which is an aging society with a rapidly growing older adults. In 2020, the population aged 60 years and older accounted for 18.70% of the province’s population, and the growth rate of the older adults was much higher than that of the total population (16).

### Data collection

PTB is a nationally notifiable disease in China, and medical professionals must report cases to the Chinese Center for Disease Control and Prevention through the China Information System for Disease Control and Prevention (CISDCP). We extracted all forms of PTB data by age group between 2005 and 2022. All PTB cases in Zhejiang Province were obtained from the National Notifiable Diseases Reporting System. This system is the core subsystem of the CISDCP, which enables real-time and direct reporting of notifiable infectious diseases by medical and health institutions. Population data for Zhejiang Province were obtained from the Tuberculosis Information Management System. The data collection and analysis were carried out between April 4, 2023, and September 27, 2023, providing a comprehensive examination of the trends in PTB cases within the specified timeframe.

### Study definitions

All patients with PTB met the National Diagnostic Criteria for Tuberculosis (WS288-2001, WS288-2008, and WS288-2017) and the Classification of Tuberculosis (WS196-2001 and WS196-2017). The classification criteria for TB were revised in 2017, and “tuberculous pleurisy” was included in the “pulmonary tuberculosis” category. These criteria were officially published in 2018, and the adjusted online reporting system started to be used in 2019 (17). Therefore, we summed the number of “tuberculous pleurisy” cases with the number of “pulmonary tuberculosis” cases from 2005 to 2018 to reduce bias due to the changes in criteria. The diagnosis of PTB is primarily based on etiological examination, combined with epidemiological history, clinical manifestations, chest imaging, relevant auxiliary examinations, and differential diagnosis, to arrive at a comprehensive analysis and diagnosis.

The proportion of older adults was used as the standard for measuring population aging. The proportion is positively associated with the degree of population aging. According to the United Nations Population Ageing and Its Social and Economic Consequences and the report of the First World Assembly on Ageing, population aging was defined as more than 10% of adults aged 60 years and older or more than 7% of adults aged 65 years and older in the total population (18). This study used the proportion of older adults (≥60 years) as a reference.

### Statistical analysis

This study aims to analysis the changing trends in the incidence of PTB in Zhejiang Province and the contribution of demographic changes to these trends through descriptive epidemiological methods. The number and notified incidence of PTB cases from 2005 to 2022 were analyzed by descriptive statistics using R software version 4.2.2. The notified incidence of PTB in different age groups was analyzed to compare trends in each age group. The aging-associated population attribution fraction (PAF) was calculated using Levin’s formula: PAF = p×(RR−1)/[p×(RR−1)+1] , where p is the proportion of the population aged 60 years and older, and RR is the notified incidence in the age group ≥60 years relative to the notified incidence in the age group ≤59 years. In addition to crude PTB notified incidence, age-adjusted notified incidence from 2006 to 2022 was calculated using the population of 2005 as a reference. The annual decline rate was calculated using the formula $(1-\sqrt[{17}]{P_{2022}/P_{2005}})\times 100\%$ , where P is disease notified incidence in the corresponding year. Rates were extrapolated to 2035 by fitting exponential linear regression models for crude and age-adjusted notified incidence from 2005 to 2022 to estimate the gap with the End TB Strategic target (the reduction in incidence in each period compared to 2015 levels).

The effect of demographic changes (population growth and age structure) and epidemiological changes (age-specific incidence) on the number of PTB cases was assessed using the method developed by Chen et al. (19). Epidemiological changes refer to changes in the number of cases caused by risk factors, prevention and control measures, socioeconomic status, and other factors that cannot be explained by demographic changes. Disease metrics from 2005 were used as a reference to calculate the absolute and relative contributions of population growth, age structure, and age-specific incidence in 2006–2022. Positive and negative contributions indicate an increase and a decrease in the number of cases, respectively. The details of the decomposition method are presented in Supplementary Methods.

## Results

### PTB notified incidence in Zhejiang Province

**Figure 1:**
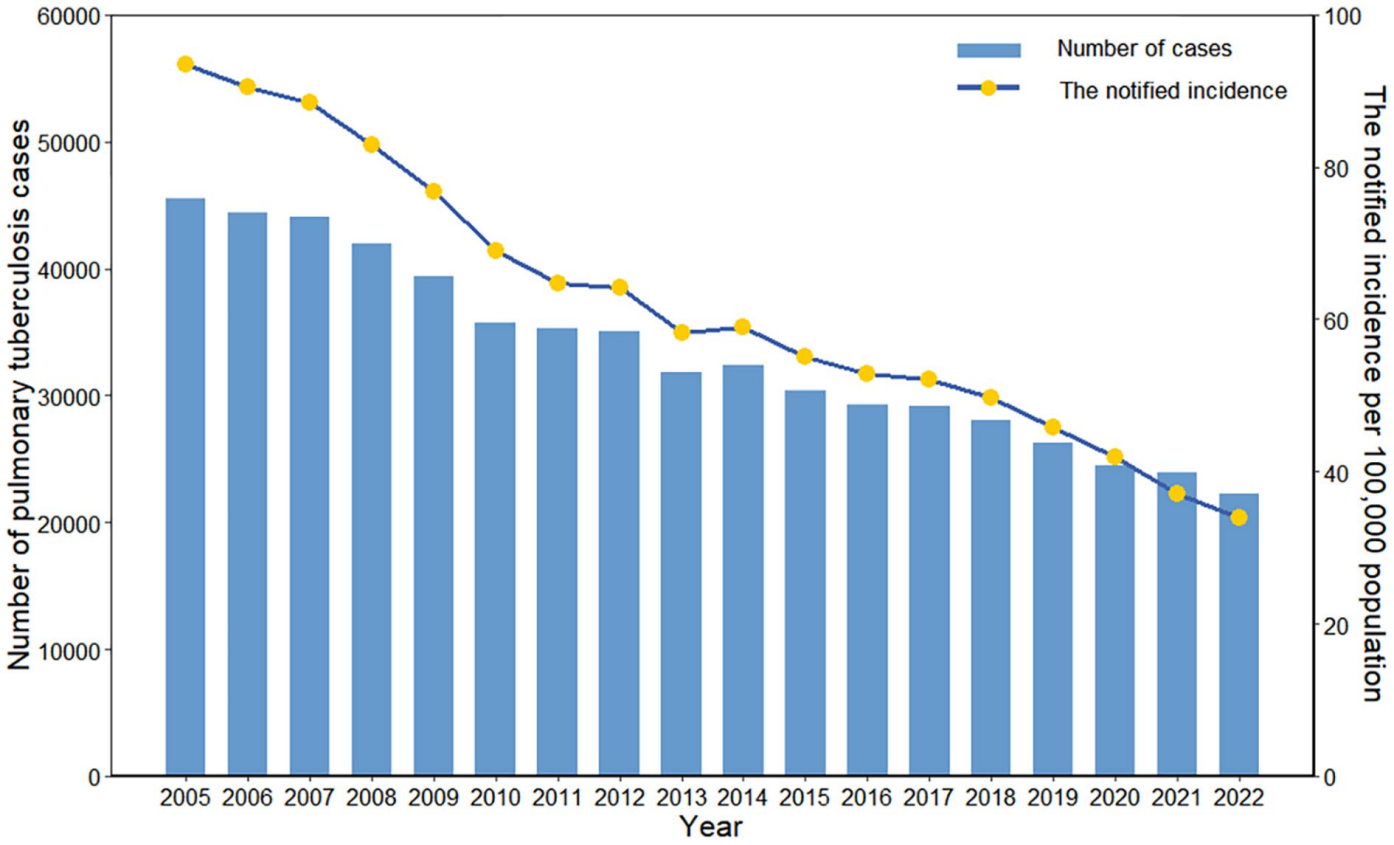
Number of cases and the notified incidence of pulmonary tuberculosis in Zhejiang Province, China.

**Figure 2:**
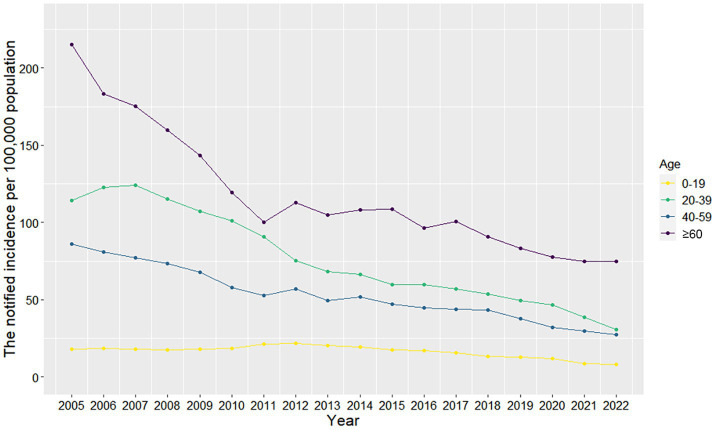
Notified incidence of pulmonary tuberculosis by age group.

A total of 599,526 cases of PTB were reported in Zhejiang Province between 2005 and 2022, and the number of new cases decreased from 45,567 to 22,227. The notified incidence of PTB decreased from 93.50 per 100,000 in 2005 to 33.99 per 100,000 in 2022, corresponding to a 5.78% annual decline (Figure 1). There were fluctuating downward trends in the notified incidence of PTB across age groups (Supplementary Figure 1). The highest notified incidence was observed among those aged 60 years and older, followed by those aged 20–39 years and 40–59 years, with the lowest notified incidence among those under 20 years of age (Figure 2).

**Figure 3:**
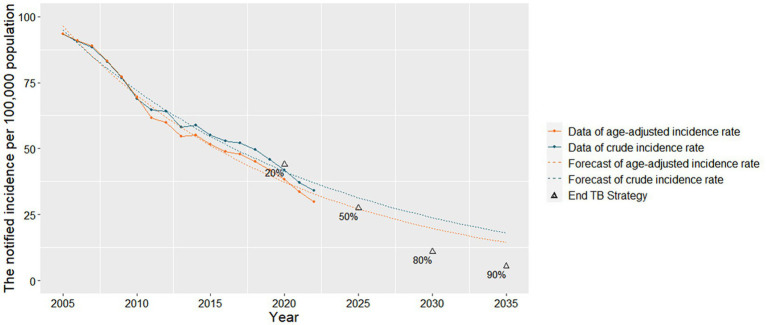
Crude and age-adjusted notified incidence of pulmonary tuberculosis in Zhejiang Province.

After exponential model fitting, total and age-adjusted notified incidence decreased from 94.95 per 100,000 and 96.44 per 100,000 in 2005 to 37.08 per 100,000 and 32.85 per 100,000 in 2022, respectively (Figure 3; Supplementary Table 1). The crude notified incidence decreased by an average of 5.38% per year, and the age-adjusted notified incidence decreased by 6.14% per year. Extrapolating this trend, the crude and age-adjusted notified incidence of PTB in 2035 is 18.07 and 14.41 per 100,000, representing a 67.19 and 73.83% decrease compared to 2015.

### Changes in PTB burden among older adults in Zhejiang Province

The total population of Zhejiang Province in 2022 was estimated to be 65.40 million, an increase of 16.66 million compared with 48.74 million in 2005, representing an increase of 34.18%, and an average annual growth of 1.74% (Supplementary Figure 2). From 2005 to 2022, 171,544 cases of PTB were reported among the population aged 60 years and older (Table 1). Despite the notified incidence of PTB decreased from 215.01 per 100,000 to 75.06 per 100,000 (Supplementary Table 2), the proportion of PTB cases among older adults increased from 29 to 41.45% of the total number of cases. This trend may be driven by changes in the demographic structure of older adults. The proportion of older adults in the total population increased from 12.61 to 18.77%. And the PAF for this age group increased from 18.76 to 27.92%.

**Table 1:** Notified incidence of pulmonary tuberculosis and population among the people aged 60 years and older in Zhejiang Province, China, 2005–2022.

Year	Number of cases	Percentage of cases (%)	Proportion of older adults (%)	Notified incidence (1/10^5^)	PAF (%)
2005	13,215	29.00	12.61	215.01	18.76
2006	11,370	25.63	12.67	183.26	14.84
2007	11,187	25.38	12.83	175.08	14.4
2008	10,579	25.19	13.08	159.79	13.93
2009	9,830	24.98	13.41	143.14	13.35
2010	8,518	23.83	13.8	119.19	11.64
2011	7,936	22.53	14.59	99.95	9.30
2012	8,771	25.00	14.26	112.62	12.53
2013	8,385	26.32	14.59	104.94	13.73
2014	8,994	27.77	15.12	108.20	14.90
2015	9,042	29.81	15.11	108.64	17.32
2016	8,705	29.69	16.28	96.53	16.02
2017	9,287	31.85	16.49	100.76	18.39
2018	9,329	33.23	18.16	90.80	18.41
2019	9,244	35.19	19.37	83.17	19.61
2020	8,887	36.24	19.51	77.86	20.79
2021	9,053	37.77	18.70	74.99	23.46
2022	9,212	41.45	18.77	75.06	27.92

PAF, population attributable fraction.

### Effect of demographic and epidemiological changes on PTB burden

**Figure 4:**
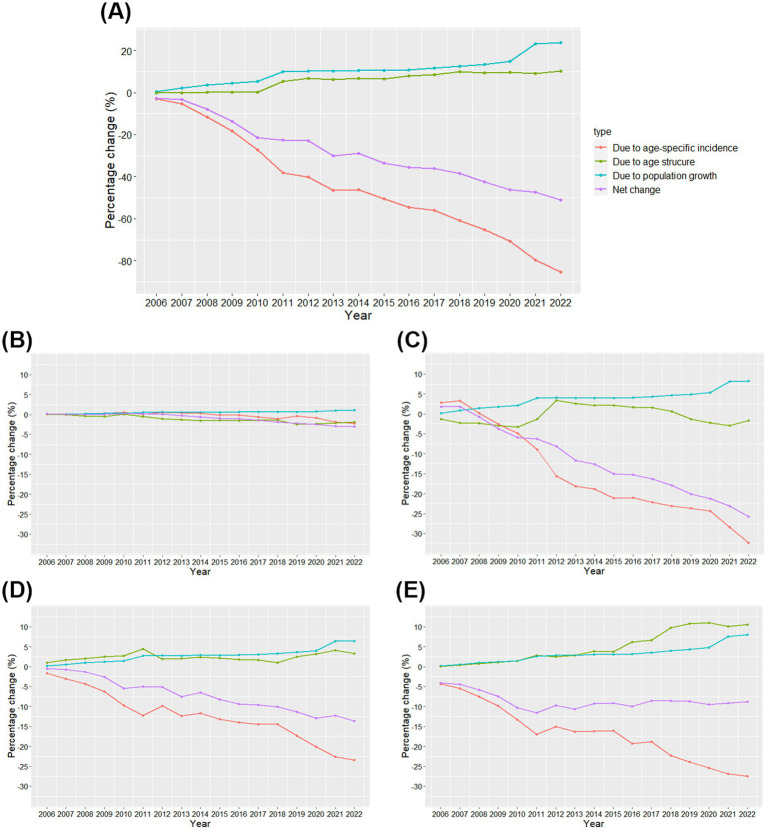
Contributions of changes in population growth, age structure, and age-specific incidence to changes in the number of cases of pulmonary tuberculosis in Zhejiang Province from 2006 to 2022 **(A)** and in the age groups 0–19, 20–39, 40–59, and ≥60 years **(B–E)**. The year 2005 was used as a reference.

Between 2005 and 2022, the number of PTB cases decreased by 51.22%, driven by a 23.83% increase due to population growth, a 10.26% increase due to age structure, and an 85.31% decrease due to age-specific incidence (Figure 4; Supplementary Table 3). In the age group 0–19 years, the number of cases decreased by 3.02% compared to 2005 due to a 1.08% increase in population growth, a 1.92% decrease in age structure, and a 2.18% decrease in age-specific incidence. In the age group 20–39 years, the number of cases decreased by 25.75% compared to 2005, mainly due to an 8.27% increase in population growth, a 1.69% decrease in age structure, and a 32.32% decrease in age-specific incidence. In the age group 40–59 years, the number of cases decreased by 13.66%, mainly attributed to a 6.45% increase in population growth, a 3.28% increase in age structure, and a 23.39% decrease in age-specific incidence. The number of cases in people aged 60 years and older decreased by 8.78% compared to 2005, driven by an 8.04% increase due to population growth, a 10.60% increase due to age structure, and a 27.42% decrease in age-specific incidence.

## Discussion

The notified incidence of PTB in Zhejiang Province showed a downward trend. However, achieving the End TB Strategy 2035 goal is challenging. Notably, the notified incidence was higher in individuals aged 60 years and older compared to those under 60. The age structure of the older adults showed a fluctuating upward trend. Epidemiological changes were the main drivers of the decline in the number of PTB cases.

In this study, the notified incidence in the older adults was consistently high. This result was consistent with other studies (20). Wang et al. reported that the highest incidence of PTB was among those aged 60 years and older in China (21). TB was also more common in older adults in countries such as the USA and Japan (5, 22). Natural aging increases the risk of lung and immune dysfunction, increasing susceptibility to lung diseases (23). Aging affects the lungs at molecular and cellular levels (24). The severity of lung inflammation increases with age (25), elevating the risk of TB reactivation and susceptibility to new infections in older adults. In addition, age-related comorbidities, including diabetes, malnutrition, and cancer, elevate the risk of TB in older adults (7).

Furthermore, our findings suggested that during the COVID-19 pandemic period (2020–2022), the notified incidence of PTB among older adults in Zhejiang Province continued to decline. However, this downward trend showed a marked flattening compared to the long-term trajectory observed prior to the pandemic (2005–2019). This shift in trend suggests that the pandemic may have had complex effects on the epidemiological burden of PTB. On the one hand, strict lockdown measures, strained medical resources, and public apprehension about seeking medical care during the early stages of the pandemic likely resulted in some older patients with mild PTB symptoms not being timely identified and treated, leading to a temporary underreporting of cases (26, 27). However, as epidemic prevention and control measures became routine, these cases were gradually identified. This consequently slowed the downward trend in the notified incidence. On the other hand, interventions such as mask-wearing and social distancing likely reduced the transmission of *Mycobacterium tuberculosis* (MTB) to some extent. This has contributed positively and sustainably to the decline in the notified incidence. Therefore, the flattening of the downward trend may represent an equilibrium achieved through the interaction between the diminishing effect of delayed diagnosis and the slowing of MTB transmission.

The proportion of PTB cases among people aged 60 years and older shifted in 2011 from a decreasing to an increasing trend in our results. This may be the result of a combination of factors that led to improved detection of TB cases in older adults. For instance, in 2011 and 2012, most counties in Zhejiang Province completed a shift in service delivery model, transferring the function of TB diagnosis and treatment to designated hospitals (28). This strategy increased the accessibility and continuity of TB services, facilitated the completion of in-hospital referrals for older adults. Moreover, health management services for patients with TB were included in the national basic public health service in 2011 (29), which has helped to strengthen the screening and management of suspected cases. Furthermore, the improved standard of living and household income of older adults increases to some extent their awareness of diseases and their healthcare-seeking and health behaviors.

Kochi et al. estimated that 79.5% of new TB cases could be attributed to population growth and changes in age structure (30). Our results indicated that the impact of age structure on changes in the number of PTB cases was different in various age groups. The contribution of changes in the age structure to the number of cases among people aged 60 and older showed a clear fluctuating upward trend. Changes in age structure influence TB risk (31, 32), and the older adults’ proportion rises, so does their TB risk (33). China is aging rapidly (34). We found that the proportion of the population aged 60 years and older accounted for more than 10% of the total population. An increase in the older adults indicated the aggravation of population aging. Therefore, we need to assess the impact of population changes on TB, strengthen TB prevention, screening, diagnosis, and treatment, increase public health awareness, and improve the allocation of healthcare resources, especially in older adults.

The Chinese population has consistently increased over the years, and population growth is positively correlated with the number of PTB cases. The increase in population growth during 2010–2011 and 2020–2021 may have been influenced by the sixth and seventh national population censuses. The population in non-census years was extrapolated from sample survey data from that year. There were biases in overall population projections because of underreporting in sample surveys in some years, and biases accumulated over time. Therefore, census data were not consistent with the trend reflected by data from non-census years. Population growth may promote the spread of PTB, leading to an increase in the number of PTB cases. Factors such as increased population density (35, 36), greater social mobility (37, 38), and inadequate healthcare facilities can contribute to easier transmission of MTB. Additionally, population growth may lead to an uneven distribution of resources, making it challenging to implement effective TB prevention and control measures, exacerbating the risk of disease transmission and progression.

TB susceptibility and severity are increased by overcrowding, indoor air pollution, smoking, alcohol consumption, malnutrition, HIV infection, and diabetes (39–41). These factors can hinder the reduction in the notified incidence of PTB. However, the notified incidence of PTB in Zhejiang Province showed a fluctuating decline in different age groups. This decline was contingent upon how other epidemiological factors—improvements in TB control, socioeconomic development, and a decrease in transmission—counterbalanced the increase attributable to population aging (42). The crucial role of social and economic development policies and interventions in supporting PTB control has gained attention in recent years. Several initiatives have enhanced PTB control, including reviewing legal documents on infectious disease control and implementing an internet-based infectious disease reporting system in 2004 (21, 43). The full implementation of the Directly Observed Treatment Short-Course Strategy in China since 2005 has significantly improved PTB detection by extending diagnosis and treatment to all patients (21). The government conducted PTB screening in the migrant population, strengthened screening in schools, and launched key screening programs for the older adults to identify and manage active PTB cases. Concurrently, a well-developed healthcare system and policies encouraged healthcare-seeking behaviors and reduced social transmission (44). The introduction of rapid molecular tests, such as GeneXpert MTB/RIF, shortened the diagnostic time (45). Moreover, the nationwide and free provision of diagnostic tests and treatment interventions for patients with TB since 2004 has greatly reduced the financial burden, increased adherence, and improved treatment outcomes (46).

The notified incidence of PTB in Zhejiang Province decreased with the effective implementation of these measures and projects. However, it remained distant from the 2035 target of the End TB Strategy (14, 47). China is nearing the 2035 mortality target but is less likely to achieve the 2035 morbidity target (15). Tools are needed to reduce screening costs for active and latent TB infections. In addition to strengthening existing measures and preferential policies (44), intensified interventions targeting key populations are required, especially for older adults. Vaccinating older adults with post-infection vaccines can significantly reduce PTB incidence and mortality (48), highlighting the need for developing such vaccines. Moreover, active screening in high-prevalence areas with low economic levels can identify more active TB cases (49), reducing community prevalence compared to passive case-finding. Preventive treatment should also be considered in regions with low incidence and environments with high transmission to eliminate latent and active TB (50).

This study is the first to quantify the effects of demographic and epidemiological changes on PTB in Zhejiang Province. We analyzed the notified incidence of PTB in Zhejiang Province as a whole and in the older adults, providing future incidence estimates. These findings have important implications for decision-making of TB prevention and control. This study has some limitations. First, considering only one province in Eastern China, regional differences may limit the generalizability of the results. Second, although we assessed the extent to which population growth, age structure, and epidemiological changes contribute to the number of PTB cases, but without further analysis of epidemiological changes. This meant that the impact of risk factors, preventive and control measures, and socio-economic status on the number of cases was not analyzed in this study. Third, constrained by the scope of surveillance data, we were unable to obtain key indicators such as patients’ treatment costs and years of healthy life lost, making it difficult to comprehensively assess the disease burden. Fourthly, the annual data used in this study limited the conduct of more refined time series analyses. Future research could utilize monthly or quarterly data to more accurately assess the impact of major public health events on disease trends. Finally, the lack of detailed individual-level demographic information prevented us from conducting more in-depth subgroup analyses. Future research should integrate multidimensional data, incorporate health economic evaluations and individual sociodemographic characteristics. This will enable precise identification of key populations and provide a foundation for developing targeted interventions.

## Conclusion

This study showed that the overall notified incidence of pulmonary tuberculosis is expected to continue to decrease in Zhejiang Province. However, achieving the End TB Strategy 2035 goal is challenging because of demographic and epidemiological changes. Population aging has shifted the burden of TB to older adults, bringing new challenges to its diagnosis and management. Therefore, reduction in the burden of TB requires additional efforts, including intensifying latent TB screening in high-risk populations, developing new diagnostic and treatment methods, and applying preventive treatment to reduce the risk of TB among people with latent TB infection.

## Data Availability

The raw data supporting the conclusions of this article will be made available by the authors, without undue reservation.
