# Supplementary material for: Effect of population aging on pulmonary tuberculosis burden in Zhejiang Province, China: a population-based study
Source: Front Public Health. 2025 Oct 29;13:1647139. doi: 10.3389/fpubh.2025.1647139 (PMC12604989; doi:10.3389/fpubh.2025.1647139)
Supplement: Supplementary file 1 [file Table_1.DOCX]

**Supplementary Material**

**Effect of Population Aging on Pulmonary Tuberculosis Burden** **in Zhejiang Province, China: A Population-Based Study**

**Table of content**

eMethods. The decomposition method.

eTable 1 Estimated crude and age-adjusted notified incidence of pulmonary tuberculosis in Zhejiang Province from 2005 to 2035.

eTable 2 Notified incidence of pulmonary tuberculosis among people under 60 years of age, 2005-2022.

eTable 3. Contribution of changes in population growth, age structure, and age-specific incidence to the change of the number of pulmonary tuberculosis cases in Zhejiang Province, from 2006 to 2022, using 2005 as the reference year.

eFigure 1 Notified incidence of pulmonary tuberculosis in different age groups.

eFigure 2 Population changes in Zhejiang Province from 2005 to 2022.

**eMethods. The decomposition method**

The population decomposition algorithm has been described in detail in the papers by Cheng et al. ^1,2^.

Briefly, take the difference in the new cases of pulmonary tuberculosis (PTB) between 2005 and 2022 in Zhejiang province. We can decompose the net change of PTB cases into the contribution of three factors: population growth, age structure, and age-specific incidence rate.

The age groups were divided using 5-year increments from 0-4 years to 85 plus (we included older people aged ≥85 years as the 85-89 years age group, which was recorded as only one group in the database).

Let *d_ij_*, *n_ij_*, *m_ij_* and *s_ij_* denote the incident cases, population size, age-specific rate of incidence, and population proportion in the *i*^th^ age group of the year *j*, respectively, (*i* = 1, 2, …,12; *j* = 1, 2). Let *D*_1_ and *D*_2_, *N*_1_ and *N*_2_, *P*_1_ and *P*_2_ represent the total incident cases, population size, and crude incidence rate in 2005 and 2022, respectively.

Using *M_p_*, *M_a,_* and *M_m_* to represent the main effects of the changes in population size, age structure, and incidence rate, and *I_pa_*, *I_pm_*, *I_am,_* and *I_pam_* to represent their two-way and three-way interactions, respectively. In the case of 2005 as the reference year, these terms are calculated as follows:

$M_{p}=\sum_{i=1}^{12} {{\left( N_{2}-N_{1} \right)s}_{i1}m}_{i1}$

$M_{a}=\sum_{i=1}^{12} N_{1}\left( s_{i2}-s_{i1} \right)m_{i1}$

$M_{m}=\sum_{i=1}^{12} {N_{1}s}_{i1}\left( m_{i2}-m_{i1} \right)$

$I_{pa}=\sum_{i=1}^{12} \left( N_{2}-N_{1} \right)\left( s_{i2}-s_{i1} \right)m_{i1}$

$I_{pm}=\sum_{i=1}^{12} \left( N_{2}-N_{1} \right)s_{i1}\left( m_{i2}-m_{i1} \right)$

$I_{am}=\sum_{i=1}^{12} N_{1}\left( s_{i2}-s_{i1} \right)\left( m_{i2}-m_{i1} \right)$

$I_{pam}=\sum_{i=1}^{12} \left( N_{2}-N_{1} \right)\left( s_{i2}-s_{i1} \right)\left( m_{i2}-m_{i1} \right)$

Here, a simplification needs to be made, assuming that the interactions are equally distributed, then the contribution of the three factors can be calculated as follows:

$A{=M}_{a}+½I_{am}+½I_{pa}+⅓I_{pam}$

$P{=M}_{p}+½I_{pm}+½I_{pa}+⅓I_{pam}$

$M{=M}_{m}+½I_{pm}+½I_{am}+⅓I_{pam}$

Here, *A* represents the contribution of age structure, *P* represents the contribution of population growth, *M* represents the contribution of the age-specific death rate, and net change represents total change. The contribution of each factor divided by *D_1_* and multiplied by 100 is the percentage of the respective contribution.

**References**

1. Cheng X, Tan L, Gao Y, Yang Y, Schwebel DC, Hu G. A new method to attribute differences in total deaths between groups to population size, age structure and age-specific mortality rate. *PLoS One*. 2019;14(5):e0216613. doi:10.1371/journal.pone.0216613

2. Cheng X, Yang Y, Schwebel DC, et al. Population ageing and mortality during 1990-2017: A global decomposition analysis. *PLoS Med*. Jun 2020;17(6):e1003138. doi:10.1371/journal.pmed.1003138

| eTable 1 Estimated crude and age-adjusted notified incidence of pulmonary tuberculosis in Zhejiang Province from 2005 to 2035. | | |
| --- | --- | --- |
| Year | Crude incidence (1/10^5^) | Age-standardized incidence (1/10^5^) |
| 2005 | 94.95 | 96.44 |
| 2006 | 89.84 | 90.52 |
| 2007 | 85.01 | 84.96 |
| 2008 | 80.43 | 79.75 |
| 2009 | 76.10 | 74.85 |
| 2010 | 72.01 | 70.25 |
| 2011 | 68.13 | 65.94 |
| 2012 | 64.47 | 61.89 |
| 2013 | 61.00 | 58.09 |
| 2014 | 57.72 | 54.53 |
| 2015 | 54.61 | 51.18 |
| 2016 | 51.67 | 48.04 |
| 2017 | 48.89 | 45.09 |
| 2018 | 46.26 | 42.32 |
| 2019 | 43.77 | 39.72 |
| 2020 | 41.42 | 37.28 |
| 2021 | 39.19 | 34.99 |
| 2022 | 37.08 | 32.85 |
| 2023 | 35.08 | 30.83 |
| 2024 | 33.20 | 28.94 |
| 2025 | 31.41 | 27.16 |
| 2026 | 29.72 | 25.49 |
| 2027 | 28.12 | 23.93 |
| 2028 | 26.61 | 22.46 |
| 2029 | 25.18 | 21.08 |
| 2030 | 23.82 | 19.78 |
| 2031 | 22.54 | 18.57 |
| 2032 | 21.33 | 17.43 |
| 2033 | 20.18 | 16.36 |
| 2034 | 19.09 | 15.36 |
| 2035 | 18.07 | 14.41 |

| eTable 2 Notified incidence of pulmonary tuberculosis among people under 60 years of age, 2005-2022. | |
| --- | --- |
| Year | Notified incidence (1/10^5^) |
| 2005 | 75.96 |
| 2006 | 77.13 |
| 2007 | 75.77 |
| 2008 | 71.42 |
| 2009 | 66.61 |
| 2010 | 60.96 |
| 2011 | 58.70 |
| 2012 | 56.18 |
| 2013 | 50.18 |
| 2014 | 50.13 |
| 2015 | 45.53 |
| 2016 | 44.45 |
| 2017 | 42.57 |
| 2018 | 40.49 |
| 2019 | 36.81 |
| 2020 | 33.20 |
| 2021 | 28.41 |
| 2022 | 24.50 |

| eTable 3 Contribution of changes in population growth, age structure, and age-specific incidence to the change of the number of pulmonary tuberculosis cases in Zhejiang Province, from 2006 to 2022, using 2005 as the reference year. | | | | |
| --- | --- | --- | --- | --- |
| Year | Due to population growth, n (%) | Due to age structure,  n (%) | Due to age-specific incidence, n (%) | Net change, n (%) |
| Total | | | | |
| 2006 | 225 (0.49) | -73 (-0.16) | -1356 (-2.98) | -1203 (-2.64) |
| 2007 | 970 (2.13) | -103 (-0.23) | -2357 (-5.17) | -1490 (-3.27) |
| 2008 | 1648 (3.62) | 58 (0.13) | -5282 (-11.59) | -3576 (-7.85) |
| 2009 | 2103 (4.62) | 70 (0.15) | -8381 (-18.39) | -6208 (-13.62) |
| 2010 | 2496 (5.48) | 104 (0.23) | -12429 (-27.28) | -9829 (-21.57) |
| 2011 | 4533 (9.95) | 2450 (5.38) | -17327 (-38.03) | -10344 (-22.70) |
| 2012 | 4680 (10.27) | 3082 (6.76) | -18243 (-40.04) | -10481 (-23.00) |
| 2013 | 4616 (10.13) | 2823 (6.20) | -21147 (-46.41) | -13707 (-30.08) |
| 2014 | 4803 (10.54) | 3124 (6.86) | -21106 (-46.32) | -13178 (-28.92) |
| 2015 | 4762 (10.45) | 3027 (6.64) | -23025 (-50.53) | -15236 (-33.44) |
| 2016 | 4940 (10.84) | 3642 (7.99) | -24830 (-54.49) | -16248 (-35.66) |
| 2017 | 5292 (11.61) | 3822 (8.39) | -25519 (-56.00) | -16405 (-36.00) |
| 2018 | 5715 (12.54) | 4549 (9.98) | -27758 (-60.92) | -17494 (-38.39) |
| 2019 | 6139 (13.47) | 4332 (9.51) | -29768 (-65.33) | -19297 (-42.35) |
| 2020 | 6765 (14.85) | 4404 (9.66) | -32215 (-70.70) | -21046 (-46.19) |
| 2021 | 10557 (23.17) | 4177 (9.17) | -36332 (-79.73) | -21598 (-47.40) |
| 2022 | 10857 (23.83) | 4677 (10.26) | -38874 (-85.31) | -23340 (-51.22) |
| 0 - 19 years old | | | | |
| 2006 | 12 (0.03) | 44 (0.10) | 28 (0.06) | 84 (0.18) |
| 2007 | 50 (0.11) | -15 (-0.03) | 5 (0.01) | 40 (0.09) |
| 2008 | 87 (0.19) | -153 (-0.34) | 44 (0.10) | -22 (-0.05) |
| 2009 | 115 (0.25) | -221 (-0.49) | 122 (0.27) | 15 (0.03) |
| 2010 | 144 (0.32) | 7 (0.02) | 261 (0.57) | 85 (0.19) |
| 2011 | 260 (0.57) | -255 (-0.56) | 56 (0.12) | 61 (0.13) |
| 2012 | 267 (0.59) | -505 (-1.11) | 256 (0.56) | 19 (0.04) |
| 2013 | 264 (0.58) | -574 (-1.26) | 167 (0.37) | -143 (-0.31) |
| 2014 | 265 (0.58) | -686 (-1.51) | 141 (0.31) | -280 (-0.61) |
| 2015 | 257 (0.56) | -661 (-1.45) | -64 (-0.14) | -467 (-1.02) |
| 2016 | 267 (0.59) | -686 (-1.51) | -90 (-0.20) | -509 (-1.12) |
| 2017 | 274 (0.60) | -660 (-1.45) | -282 (-0.62) | -667 (-1.46) |
| 2018 | 283 (0.62) | -669 (-1.47) | -484 (-1.06) | -870 (-1.91) |
| 2019 | 302 (0.66) | -1128 (-2.48) | -187 (-0.41) | -1014 (-2.23) |
| 2020 | 326 (0.72) | -1061 (-2.33) | -382 (-0.84) | -1116 (-2.45) |
| 2021 | 475 (1.04) | -970 (-2.13) | -838 (-1.84) | -1333 (-2.93) |
| 2022 | 490 (1.08) | -876 (-1.92) | -992 (-2.18) | -1378 (-3.02) |
| 20 - 39 years old | | | | |
| 2006 | 91 (0.20) | -612 (-1.34) | 1318 (2.89) | 797 (1.75) |
| 2007 | 393 (0.86) | -1043 (-2.29) | 1478 (3.24) | 829 (1.82) |
| 2008 | 661 (1.45) | -1058 (-2.32) | 61 (0.13) | -335 (-0.74) |
| 2009 | 836 (1.83) | -1331 (-2.92) | -1191 (-2.61) | -1686 (-3.70) |
| 2010 | 1001 (2.20) | -1511 (-3.32) | -2212 (-4.85) | -2722 (-5.97) |
| 2011 | 1813 (3.98) | -591 (-1.3) | -4065 (-8.92) | -2842 (-6.24) |
| 2012 | 1845 (4.05) | 1574 (3.45) | -7121 (-15.63) | -3701 (-8.12) |
| 2013 | 1799 (3.95) | 1169 (2.57) | -8271 (-18.15) | -5304 (-11.64) |
| 2014 | 1833 (4.02) | 1002 (2.20) | -8554 (-18.77) | -5719 (-12.55) |
| 2015 | 1797 (3.94) | 956 (2.10) | -9621 (-21.11) | -6868 (-15.07) |
| 2016 | 1872 (4.11) | 744 (1.63) | -9594 (-21.05) | -6978 (-15.31) |
| 2017 | 1982 (4.35) | 691 (1.52) | -10092 (-22.15) | -7419 (-16.28) |
| 2018 | 2104 (4.62) | 287 (0.63) | -10528 (-23.10) | -8138 (-17.86) |
| 2019 | 2217 (4.87) | -589 (-1.29) | -10777 (-23.65) | -9149 (-20.08) |
| 2020 | 2435 (5.34) | -1013 (-2.22) | -11125 (-24.41) | -9702 (-21.29) |
| 2021 | 3725 (8.17) | -1321 (-2.90) | -12924 (-28.36) | -10520 (-23.09) |
| 2022 | 3768 (8.27) | -772 (-1.69) | -14729 (-32.32) | -11733 (-25.75) |
| 40 - 59 years old | | | | |
| 2006 | 61 (0.13) | 444 (0.97) | -744 (-1.63) | -239 (-0.52) |
| 2007 | 262 (0.57) | 782 (1.72) | -1375 (-3.02) | -331 (-0.73) |
| 2008 | 452 (0.99) | 944 (2.07) | -1978 (-4.34) | -583 (-1.28) |
| 2009 | 580 (1.27) | 1117 (2.45) | -2850 (-6.25) | -1152 (-2.53) |
| 2010 | 680 (1.49) | 1252 (2.75) | -4427 (-9.72) | -2495 (-5.48) |
| 2011 | 1257 (2.76) | 2030 (4.45) | -5570 (-12.22) | -2284 (-5.01) |
| 2012 | 1281 (2.81) | 868 (1.90) | -4505 (-9.89) | -2355 (-5.17) |
| 2013 | 1256 (2.76) | 930 (2.04) | -5616 (-12.32) | -3430 (-7.53) |
| 2014 | 1325 (2.91) | 1061 (2.33) | -5344 (-11.73) | -2958 (-6.49) |
| 2015 | 1303 (2.86) | 994 (2.18) | -6025 (-13.22) | -3728 (-8.18) |
| 2016 | 1332 (2.92) | 793 (1.74) | -6376 (-13.99) | -4251 (-9.33) |
| 2017 | 1421 (3.12) | 763 (1.67) | -6574 (-14.43) | -4391 (-9.64) |
| 2018 | 1528 (3.35) | 467 (1.02) | -6595 (-14.47) | -4600 (-10.10) |
| 2019 | 1643 (3.61) | 1114 (2.44) | -7920 (-17.38) | -5163 (-11.33) |
| 2020 | 1798 (3.95) | 1448 (3.18) | -9147 (-20.07) | -5900 (-12.95) |
| 2021 | 2894 (6.35) | 1849 (4.06) | -10326 (-22.66) | -5583 (-12.25) |
| 2022 | 2937 (6.45) | 1494 (3.28) | -10658 (-23.39) | -6226 (-13.66) |
| ≥ 60 years old | | | | |
| 2006 | 62 (0.14) | 51 (0.11) | -1958 (-4.30) | -1845 (-4.05) |
| 2007 | 264 (0.58) | 173 (0.38) | -2465 (-5.41) | -2028 (-4.45) |
| 2008 | 448 (0.98) | 324 (0.71) | -3409 (-7.48) | -2636 (-5.78) |
| 2009 | 573 (1.26) | 505 (1.11) | -4463 (-9.79) | -3385 (-7.43) |
| 2010 | 671 (1.47) | 682 (1.50) | -6051 (-13.28) | -4697 (-10.31) |
| 2011 | 1203 (2.64) | 1266 (2.78) | -7748 (-17.00) | -5279 (-11.59) |
| 2012 | 1286 (2.82) | 1143 (2.51) | -6873 (-15.08) | -4444 (-9.75) |
| 2013 | 1297 (2.85) | 1299 (2.85) | -7426 (-16.30) | -4830 (-10.60) |
| 2014 | 1380 (3.03) | 1747 (3.83) | -7348 (-16.13) | -4221 (-9.26) |
| 2015 | 1404 (3.08) | 1738 (3.81) | -7315 (-16.05) | -4173 (-9.16) |
| 2016 | 1469 (3.22) | 2791 (6.13) | -8769 (-19.24) | -4510 (-9.90) |
| 2017 | 1616 (3.55) | 3027 (6.64) | -8571 (-18.81) | -3928 (-8.62) |
| 2018 | 1800 (3.95) | 4464 (9.80) | -10150 (-22.27) | -3886 (-8.53) |
| 2019 | 1978 (4.34) | 4935 (10.83) | -10884 (-23.89) | -3971 (-8.71) |
| 2020 | 2205 (4.84) | 5029 (11.04) | -11562 (-25.37) | -4328 (-9.50) |
| 2021 | 3463 (7.60) | 4620 (10.14) | -12245 (-26.87) | -4162 (-9.13) |
| 2022 | 3662 (8.04) | 4831 (10.60) | -12495 (-27.42) | -4003 (-8.78) |


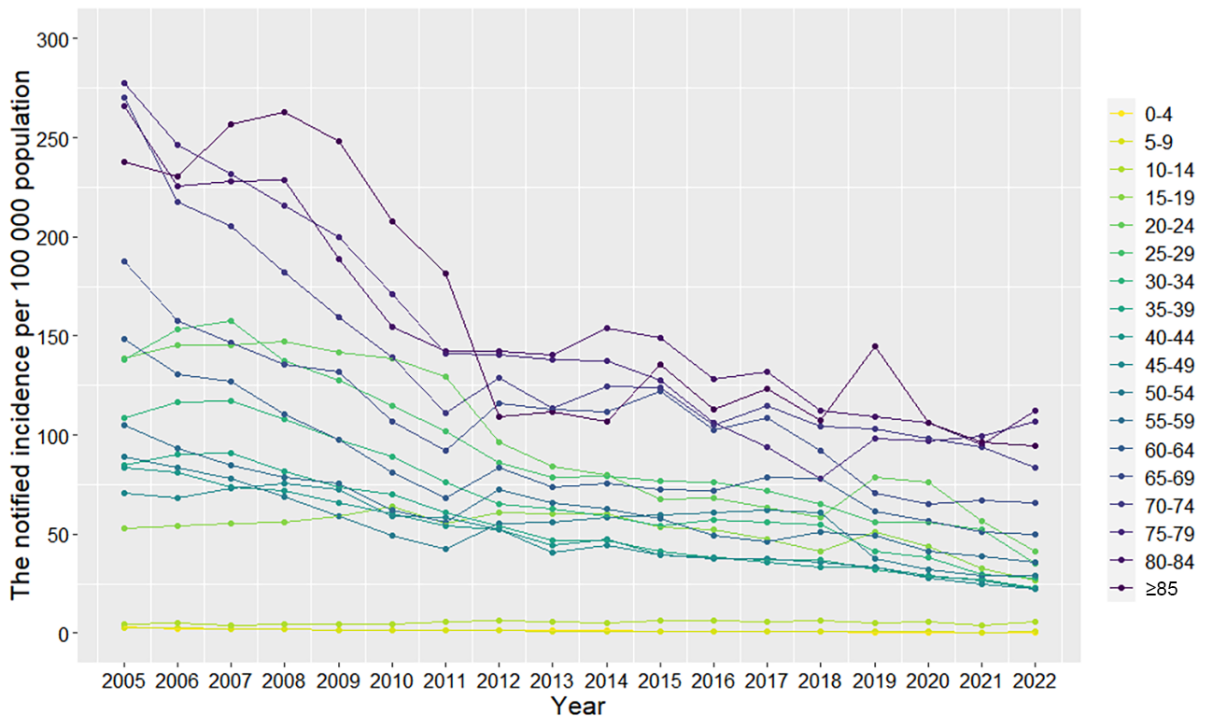


eFigure 1 Notified incidence of pulmonary tuberculosis in different age groups.


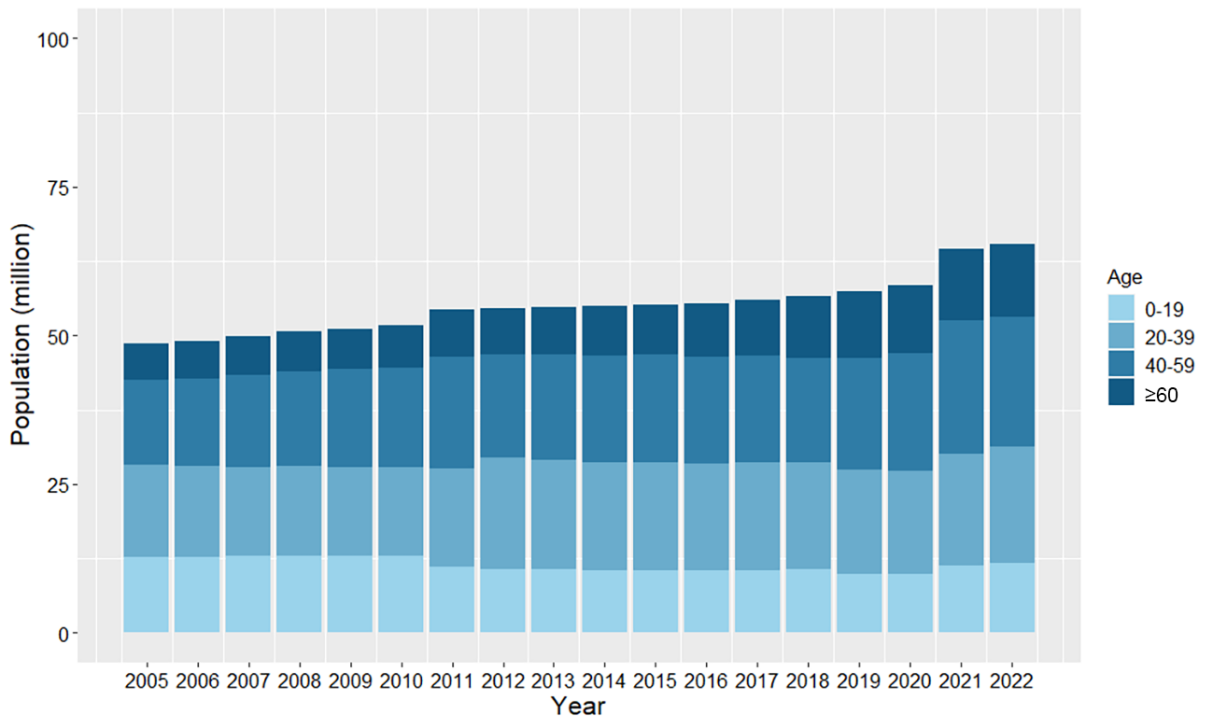


eFigure 2 Population changes in Zhejiang Province from 2005 to 2022.
